# miR-26a-Targeting SLC7A11 Regulates Erastin-Induced Granulosa Cell Ferroptosis

**DOI:** 10.3390/antiox14111283

**Published:** 2025-10-26

**Authors:** Xue Zhao, Yuheng Pan, Shuang Liang, Yuhang Lei, Yan Wang, Lei Chen, Ye Zhao, Mailin Gan, Linyuan Shen, Xin Yang, Li Zhu

**Affiliations:** 1 Animal Disease Prevention and Green Development Key Laboratory of Sichuan Province, College of Life Sciences, Sichuan University, Chengdu 610065, China; zhaoxue@stu.sicau.edu.cn; 2 Farm Animal Germplasm Resources and Biotech Breeding Key Laboratory of Sichuan Province, Sichuan Agricultural University, Chengdu 611130, China; panyuheng@stu.sicau.edu.cn (Y.P.); leiyuhang@stu.sicau.edu.cn (Y.L.); 14916@sicau.edu.cn (Y.W.); chenlei815918@sicau.edu.cn (L.C.); zhye@sicau.edu.cn (Y.Z.); ganmailin@sicau.edu.cn (M.G.); shenlinyuan@sicau.edu.cn (L.S.); 3 State Key Laboratory of Swine and Poultry Breeding Industry, College of Animal Science and Technology, Sichuan Agricultural University, Chengdu 611130, China; 4 Key Laboratory of Livestock and Poultry Multiomics, Ministry of Agriculture and Rural Affairs, College of Animal and Technology, Sichuan Agricultural University, Chengdu 611130, China

**Keywords:** granulosa cells, ferroptosis inducer, oxidantive stress, mitochondria, miRNA

## Abstract

Granulosa cell ferroptosis is a critical factor in follicular atresia and premature ovarian insufficiency (POI). As a regulated form of programmed cell death, ferroptosis is gaining significant attention in reproductive medicine research. MicroRNAs (miRNAs) play a crucial role in regulating key aspects of ferroptosis, including the glutathione-GPX4 pathway, glutamate/cystine transport, and iron and lipid metabolism. The present study demonstrates that miR-26a positively modulates ferroptosis by targeting SLC7A11, a member of the solute carrier family. We found that oocytes and granulosa cells are susceptible to the ferroptosis inducer erastin, and employed RNA sequencing to delineate the miRNA expression profiles during erastin-induced damage and ferroptosis. Notably, miR-26a expression was significantly upregulated in erastin-treated oocytes. Importantly, overexpression of miR-26a promoted ferroptosis in granulosa cells, while its knockdown inhibited this process. Ectopic miR-26a expression suppressed SLC7A11, thereby increasing ferroptosis. Our findings indicate that miR-26a influences ferroptosis by inhibiting glutathione synthesis, reducing cellular antioxidant capacity, and suggesting a potential strategy to enhance reproductive potential.

## 1. Introduction

Approximately 99% of follicles in mammals undergo a degenerative process termed atresia, which is a significant contributor to reduced female fertility [1]. Follicular atresia is a complex phenomenon, primarily characterized by the programmed cell death of granulosa cells (GCs). This association between granulosa cell death and follicular atresia has prompted extensive research into the mechanisms of granulosa cell death, in particular genomic DNA fragmentation through apoptosis [2,3]. Additionally, oxidative stress in ovarian GCs contributes to abnormal follicular development and impaired ovulation in polycystic ovary syndrome (PCOS) [4]. Ferroptosis is a form of programmed cell death characterized by iron-dependent lipid peroxidation. Emerging evidence suggests that GCs ferroptosis can significantly reduce ovarian reserve function, and is a key factor contributing to follicular atresia and premature ovarian insufficiency (POI) [5]. Excess iron promotes ROS production, inducing oxidative stress and triggering the Fenton reaction to generate hydroxyl radicals, which in turn drive lipid peroxidation and ultimately ferroptosis [6]. Glutathione peroxidase 4 (GPX4) is a crucial regulator of lipid peroxidation metabolism, functioning through its dependence on reduced glutathione (GSH) [7]. The cystine/glutamate antiporter system (Xc−system) and GSH synthesis mitigate the accumulation of lipid peroxides, thereby safeguarding cells against oxidative stress-induced ferroptosis [8]. The Xc-system is a sodium-independent countertransporter protein comprising the light chain subunit SLC7A11 and the heavy chain subunit SLC3A2. SLC7A11 drives the Xc-system’s primary role in importing cystine for glutathione (GSH) synthesis and antioxidant functions [9]. Studies have shown that SLC7A11 is overexpressed in various cancer tissues, enhancing GSH biosynthesis and promoting tumor growth by inhibiting ferroptosis [10]. Erastin is a potent inducer of ferroptosis, characterized by a distinct chemical structure featuring chlorophenoxy and quinazolinol moieties crucial for its activity. These moieties facilitate its selective binding to target proteins, thereby eliciting biological responses. Erastin functions by impeding cystine uptake in cells through the promotion of voltage-dependent anion channel (VDAC) oxidation and the inhibition of specific subunits of cystine/glutamate antiporter proteins [11,12]. Consequently, this process diminishes cellular antioxidant capacity, leading to an accumulation of reactive oxygen species (ROS) and culminating in ferroptosis. Recent studies indicate that ferroptosis plays a role in ovarian hormone synthesis and the endometrial response to hormones by regulating oxidative stress in ovarian granulosa cells [13]. GCs in atretic follicles may undergo ferroptosis when exposed to oxidative stress, iron overload, or glutathione depletion [4]. During follicular atresia, oxidative stress is prevalent and can trigger ferroptosis, hastening follicular degeneration [14].

microRNAs (miRNAs) are a class of short, non-coding RNA molecules 21–25 nucleotides in length, bind to RNA-induced silencing complexes at the 3′ untranslated regions of target mRNAs, thereby inhibiting translation initiation and subsequent protein synthesis [15]. Numerous studies have identified a strong link between ferroptosis and miRNAs. miRNAs modulate key pathways involved in ferroptosis, such as those governing mitochondrial protein expression, iron and glutathione metabolism, and lipid peroxidation. For instance, miR-144-3p can trigger ferroptosis by downregulating ZEB1 expression, thus inhibiting the proliferation, migration, and invasion of osteosarcoma cells [16]. miR-137 has been shown to negatively regulate ferroptosis in melanoma cells by directly targeting the glutamine transporter SLC1A5 [17]. Similarly, miR-93-5p promotes apoptosis and ferroptosis in gastric cancer cells by modulating the NF-κB signaling pathway [18]. Additionally, miR-5096 has been reported to induce ferroptosis in human breast cancer cells by regulating the SLC7A11/xCT signaling axis [19]. Notably, SLC7A11 serves as a marker gene for ferroptosis, and miR-26a has been demonstrated to target SLC7A11 in this process [20]. These studies tentatively suggest that miR-26a may act as a regulator of ferroptosis, but its precise molecular mechanism remains unclear.

This study investigated the role of miR-26a/SLC7A11 in erastin-induced ferroptosis. We established a connection between miR-26a/SLC7A11 and ferroptosis, elucidated the molecular mechanism of miR-26a by directly targeting SLC7A11, and delineated how miR-26a facilitates erastin-induced ferroptosis by suppressing SLC7A11 expression. This comprehensive evidence chain enhances our understanding of the regulatory network governing follicular development. Furthermore, our findings suggest that miR-26a or SLC7A11 could serve as potential biomarkers for diagnosing follicular developmental disorders such as polycystic ovary syndrome and premature ovarian failure.

## 2. Materials and Methods

### 2.1. Preparation of Sequencing Samples and RNA Sequencing

The porcine follicles were sourced from an abattoir in Chengdu, Sichuan Province, and transported to the laboratory within 1 h post-slaughter. The ovaries were rinsed twice with 1× PBS containing 1% penicillin/streptomycin (C0222, Beyotime Bio, Shanghai, China). Follicles, 3–6 mm in diameter, were selected and washed twice with DMEM/F12 (Gibco, Waltham, MA, USA) medium supplemented with 1% penicillin/streptomycin. Follicles were then cultured in DMEM/F12 medium at 37 °C with 5% CO_2_. The control group received 1 μL DMSO per ml of medium, while the experimental group was treated with 1 μL erastin (HY-15763, MedChemExpress (MCE), Monmouth Junction, New Jersey, USA) per ml of medium at 10 μM for 24 h. Treated follicles were blotted with filter paper to remove the medium, placed in pre-cooled freezing tubes, and stored at −80 °C.

### 2.2. RNA Sequencing

Total RNA was extracted from follicles and sent to Novogene Biotechnology (Beijing, China) for library construction using the Illumina Small RNA Library Preparation Kit and the Illumina TruSeq RNA Library Preparation Kit. The libraries were quality-checked and sequenced on the Illumina NovaSeq 6000 platform. Raw sequencing data underwent quality control via FastQC, alignment with Bowtie, and miRNA identification and quantification using miRDeep2. Differentially expressed miRNAs were identified based on a log2 fold change threshold of >1 and an adjusted *p*-value < 0.05. Transcriptome raw data were aligned using HISAT2 and analyzed by DESeq2 for differential gene expression. Functional annotation of differentially expressed genes and miRNAs by Gene Ontology (GO) and Kyoto Encyclopediaof Genesand Genomes (KEGG) pathway analysis.

### 2.3. Cell Culture

The mouse granule cell line (mGCs) was purchased from Pricells (Wuhan, China). The granular cells were cultured in DMEM medium (Gibco) containing 10% (*v*/*v*) fetal bovine serum (FBS, ExCell Bio, Suzhou, China) and 1% (*v*/*v*) penicillin/streptomycin (C0222, Beyotime Bio). When cell fusion was observed to 80–90%, cells were digested with trypsin (Gibco) and seeded at a density of 10 × 10^4^ cells per mL in 12-well culture plates. Experimental treatments commenced when cell density reached 80%. The cells were then exposed to 10 μM Erastin for 24 h. All cells were cultured in a cell culture incubator at 37 °C, 5% CO_2_.

### 2.4. Cell Viability Assay

The Cell Counting Kit-8 (CCK-8, CB101, Oriscience Bio, Chengdu, China) assay was employed to evaluate cell proliferation. Mouse granulocytes, ranging from 200 to 500 cells, were seeded in 96-well plates and exposed to erastin for 24 h as per the experimental protocol. Subsequently, 10 μL of CCK-8 reagent was added to each well containing 100 μL of culture medium, and the plates were incubated for an additional 2 h. Absorbance at 450 nm was measured using a spectrophotometer, and the cell proliferation inhibition rate was determined.

### 2.5. Western Blot

Cell lysates were prepared by resuspending 1 × 10^6^ cells in 200 µL of Radio Immunoprecipitation Assay (RIPA, P0013B, Beyotime Bio) buffer containing Phenylmethanesulfonyl fluoride (PMSF, ST505, Beyotime) and phosphatase inhibitors. Post-centrifugation at 12,000× *g* for 10 min, protein concentration was measured with a Bicinchoninic Acid Assay (BCA, P0010, Beyotime Bio) kit. Proteins were diluted in 5× Sodium dodecyl sulfate (SDS) buffer (P0015, Beyotime Bio), boiled at 100 °C for 10 min, and subjected to SDS-PAGE. The proteins were transferred onto a membrane via the wet-transfer method, incubated with a primary antibody overnight at 4 °C, followed by a secondary antibody for 1 h. Protein bands were visualized using Enhanced Chemiluminescence (ECL) reagent (SB-WB011, Share-Bio, Hangzhou, China) and analyzed for gray values with ImageJ software (ImageJ 1.54d).

### 2.6. RNA Extraction and Quantitative Reverse Transcription PCR (RT-qPCR)

Total RNA was extracted from follicular tissues and cells using RNAiso (TaKaRa, Dalian, China) following the manufacturer’s instructions. cDNA synthesis was conducted using the PrimeScript RT kit (TaKaRa, Dalian, China). qRT-PCR was performed on a CFX96 Real-Time Quantitative PCR System, utilizing the SYBR Premix Ex Taq II (2×) kit (TaKaRa, Dalian, China), in accordance with the provided protocol. Primers were designed with Primer 6.0 software. β-actin (ACTB) served as the internal reference for mRNA, while U6 was used for miRNA. Relative expression levels were determined using the 2^−∆∆Ct^ method. Primer sequences are listed in Table S1 (Supplementary).

### 2.7. EdU (5-Ethynyl-2′-Deoxyuridine) Staining, Mitotracker Immunofluorescence Staining and Ferr Orange Immunofluorescence Staining

Cell proliferation was assessed using an EdU staining kit (Ribobio, Guangzhou, China). Following a 24 h cell transfection, 10 μM EdU was added and incubated for 2 h. Staining followed the manual’s instructions, and images were captured via fluorescence microscopy and analyzed with ImageJ software. Mitochondrial fluorescence staining utilized the MitoTracker Red CMXRos Fluorescent Probe (C1049B, Beyotime Bio), diluted in serum-free medium to 50–200 nM, and applied to cell cultures. After 15–30 min of incubation at 37 °C, cells were washed twice with pre-warmed 1× PBS, followed by fluorescence microscopy for observation and imaging. A 1 mmol/L Ferro Orange stock solution was prepared by dissolving 24 μg of Ferro Orange in 35 μL DMSO. This stock was diluted with serum-free medium to create a 1 mmol/L working solution. After a 30 min incubation, cells were observed under a fluorescence microscope and imaged.

### 2.8. Statistical Analysis

All data were analyzed using GraphPad Prism software (GraphPad Prism 9) and presented as mean ± standard deviation (Average ± SD). A Student’s *t*-test assessed significant differences between group means, with significance defined as *p* ≤ 0.05.

## 3. Results

### 3.1. Erastin Induces Ferroptosis in Follicular and Granulosa Cells

**Figure 1:**
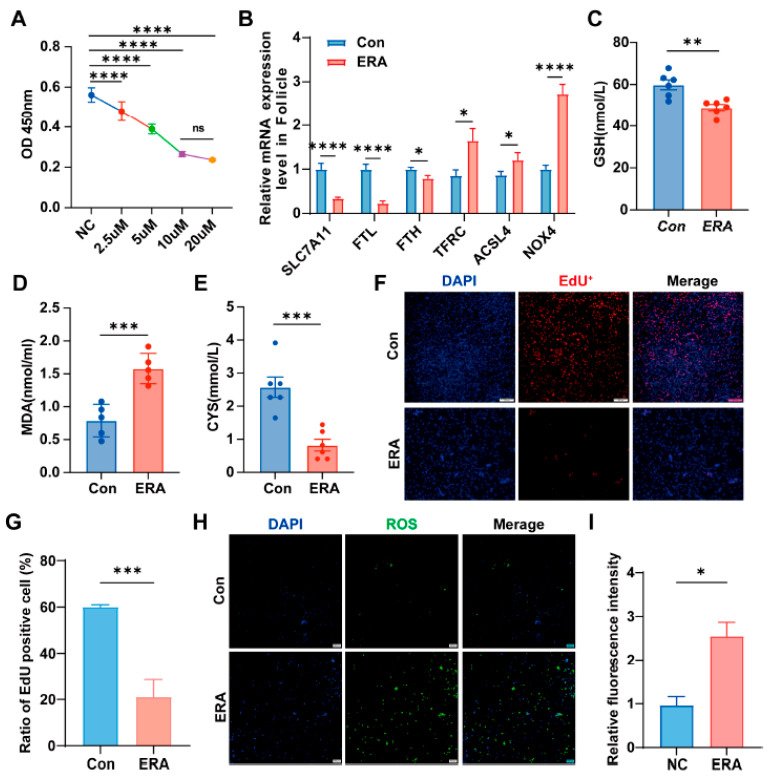
Follicle and granular cells were sensitive to erastin. (**A**) erastin concentration gradient; (**B**) Expression of ferroptosis marker gene in follicles treated with erastin; (**C**–**E**) The content of GSH, MDA and CYS in granule cells in control group (Con) and treatment group (ERA); (**F**,**G**) The EdU assay was employed to assess cell proliferation (**F**) and quantify EdU-positive cells (**G**) in both control and erastin-treated groups.; (**H**,**I**) Reactive oxygen species (ROS) were detected using ROS staining (**H**), with fluorescence intensity quantified (**I**) for the control and erastin groups. Data represented the mean ± SEM of ≥3 experiments. All *p* values were calculated using two-tailed unpaired Student’s *t* test; * *p* < 0.05, ** *p* < 0.01, *** *p* < 0.001, **** *p* < 0.001.

Previous studies have linked follicular atresia to ferroptosis. To evaluate the sensitivity of follicles and granulosa cells to erastin, granulosa cells were exposed to varying erastin concentrations. The results indicated that a 10 µM concentration inhibited cell proliferation after 24 h of treatment (Figure 1A). Subsequently, we treated the follicles with 10 µM erastin (ERA) for 24 h to assess ferroptosis marker genes. This treatment decreased the expression of SLC7A11, FTH, and FTL, while increasing TFRC, ACSL4, and NOX4 levels (Figure 1B). Our findings indicate that erastin exposure significantly decreases glutathione (GSH) (Figure 1C) and cysteine (CYS) (Figure 1E) levels in granulosa cells, while increasing malondialdehyde (MDA) (Figure 1D) levels compared to control group (Con). These results indirectly suggest that granulosa cells are susceptible to the effects of erastin. Further analysis revealed that erastin exposure led to a reduction in the number of EdU-positive cells (Figure 1F,G) and an elevation in ROS levels (Figure 1H,I) in granulosa cells.

### 3.2. miR-26a Is Highly Expressed in Erastin-Induced Ferroptosis in Follicular and Granulosa Cells

**Figure 2:**
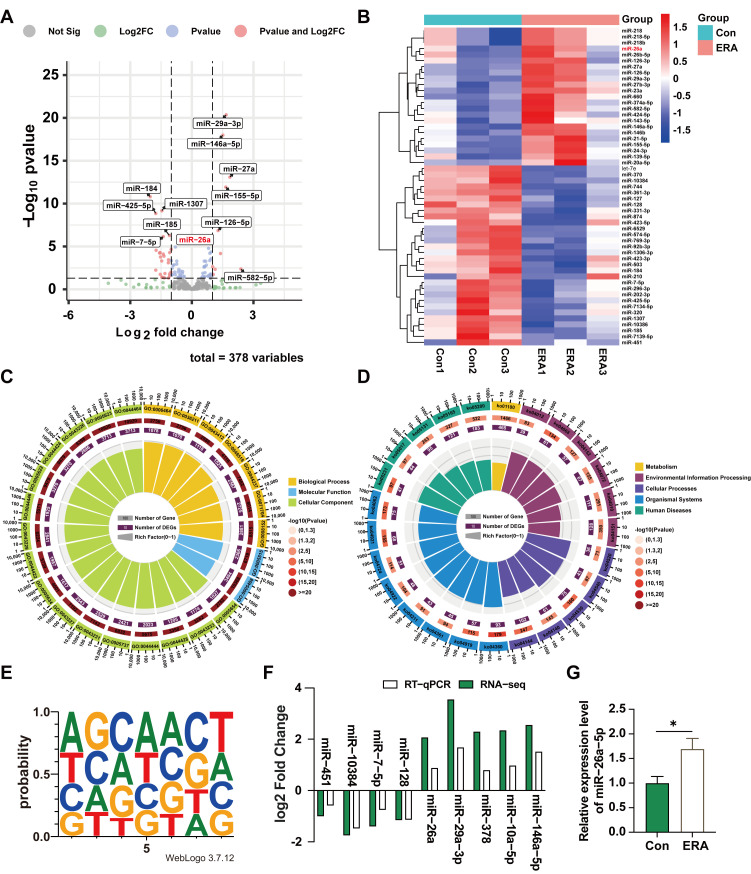
Analysis of miRNA differential expression in erastin-treated follicular and granulosa cells. (**A**) Sequencing data variance miRNAs volcano map. (**B**) miRNAs clustering heat map of sequencing data differences; (**C**) Sequencing differential miRNAs GO functional enrichment analysis; (**D**) Enrichment analysis of sequencing differential miRNAs KEGG pathway; (**E**) The up-regulated differential miRNAs seed sequences were sequenced; (**F**) qRT-PCR results verified the sequencing data; (**G**) Expression of miR-26a in granular cells treated with erastin. Data are shown as mean ± SD from at least three independent experiments. * *p* < 0.05, compared to control.

miRNAs modulate ferroptosis by regulating gene expression. To investigate differential miRNA expression between follicles and granulosa cells under the influence of the ferroptosis inducer erastin, healthy follicles treated with erastin were subjected to sequencing analysis. Differential expression analysis identified 10 upregulated and 26 downregulated miRNAs (*p* < 0.05) (Figure 2A), which were further explored through clustering analysis (Figure 2B). Functional annotation and pathway enrichment analyses were conducted to elucidate the roles of the differentially expressed miRNAs. Gene Ontology (GO) and Kyoto Encyclopedia of Genes and Genomes (KEGG) analyses revealed that the Biological Process (BP) category was primarily associated with single-organism processes, cellular processes, metabolic processes, biological regulation, and the regulation of biological processes. In the Cell Component (CC) category, the focus was on cell, cell part, organelle, and organelle part. The Molecular Function (MF) category emphasized binding, catalytic activity, and molecular function regulation (Figure 2C). KEGG pathway enrichment analysis of differential genes revealed that these enriched functions were concentrated in metabolic pathways, and the FoxO, MAPK, and Hippo signaling pathways (Figure 2D).

In addition, we analyzed nucleic acid bias of upregulated miRNAs at seed sequence locations (Figure 2E). To validate our sequencing results, we used RT-qPCR to randomly assess the expression levels of nine miRNAs. The RT-qPCR results mirrored the RNA-seq trends (Figure 2F). Notably, miR-26a was highly and differentially expressed among the up-regulated miRNAs and is conserved in pigs and mice. Furthermore, treating granulosa cells with erastin revealed that miR-26a expression remained elevated in treated cells compared to controls (Figure 2G). These findings indicate that miR-26a is highly expressed in follicular and granulosa cells undergoing ferroptosis induced by ferroptosis inducers.

### 3.3. miR-26a Suppresses Cell Proliferation, Facilitating Ferroptosis and Impairing Granulosa Cell Function

**Figure 3:**
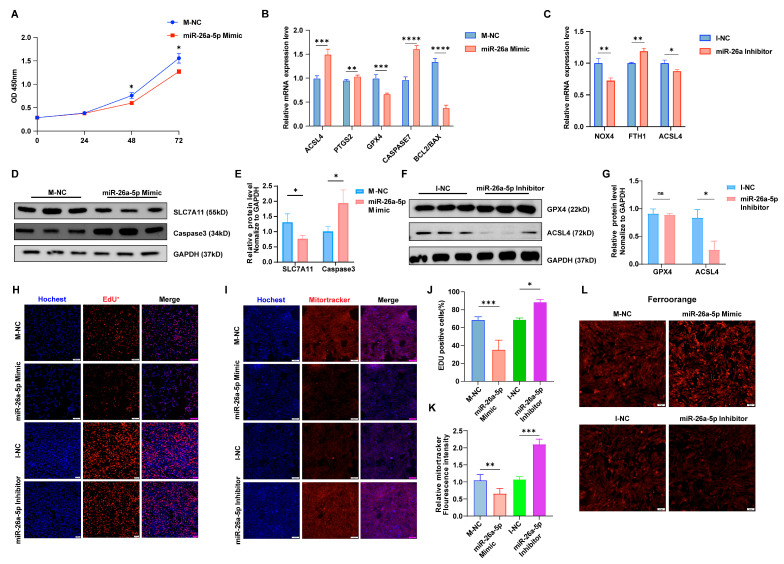
miR-26a promotes ferroptosis in granular cells. The result of CCK-8 (**A**) is consistent with the result of EdU staining assay and quantification of EUD fluorescence (**H**,**J**); Magnification, ×100. (**B**,**C**) Ferroptosis marker gene expression following miR-26a overexpression or knockdown was assessed via qRT-PCR. (**D**–**G**) Western blot analysis evaluated the expression of ferroptosis marker genes after miR-26a modulation. (**I**,**K**) Mitotracker staining assessed mitochondrial activity in both groups with miR-26a overexpression or knockdown, alongside their respective controls (**I**) and quantification of mitotracker fluorescence (**K**); Magnification, ×100. (**L**) Iron ion probe content in the control, overexpression, and knockdown groups of miR-26a was determined by iron ion staining assay; Magnification, ×100. Data are shown as mean ± SD from at least three times biological replicates. All *p*-values were calculated using a Student’s *t*-test; * *p* < 0.05, ** *p* < 0.01, *** *p* < 0.001, **** *p* < 0.0001.

To further elucidate the function of miR-26a in granulosa cells, we either overexpressed or inhibited this microRNA. Treatment with miR-26a mimics notably decreased cell viability and proliferation after 48 h (Figure 3A). Additionally, miR-26a has been shown to be highly expressed during erastin-induced ferroptosis. Therefore, the current study further examined the effect of miR-26a on cellular ferroptosis in granulosa cells. As expected, overexpression of miR-26a (miR-26a Mimic) led to an increased incidence of ferroptosis, indicated by upregulated ACSL4 and PTGS2, downregulated SLC7A11, elevated caspase7, and reduced BCL2/BAX compared to control group (M-NC) (Figure 3B). Western blot analysis confirmed a significant decrease in SLC7A11 protein levels and an increase in caspase3 expression following miR-26a overexpression (Figure 3D,E). Conversely, miR-26a knockdown (miR-26a Inhibitor) produced the opposite effects compared to negative control (I-NC) (Figure 3C,F,G). In addition, we conducted EdU staining to assess cell proliferation and observed a reduction in EdU-positive cells following miR-26a mimic treatment (Figure 3H,J). Mitotracker staining indicated that miR-26a overexpression led to decreased Mitotracker fluorescence, reflecting reduced mitochondrial activity (Figure 3I,K). Subsequently, Intracellular ferrous ion levels were measured using the probe Ferroorange, revealing that ectopic expression of miR-26a significantly elevated intracellular ferrous ion levels (Figure 3L). Conversely, miR-26a knockdown promoted cell proliferation (Figure 3H,J), enhanced mitochondrial membrane activity (Figure 3I,K), and reduced intracellular ferrous ion levels (Figure 3L). These findings indicate that miR-26a fosters a pro-apoptotic and ferroptotic environment in granulosa cells, adversely impacting their function.

### 3.4. miR-26a Is Involved in Ferroptosis by Decreasing SLC7A11 Expression

**Figure 4:**
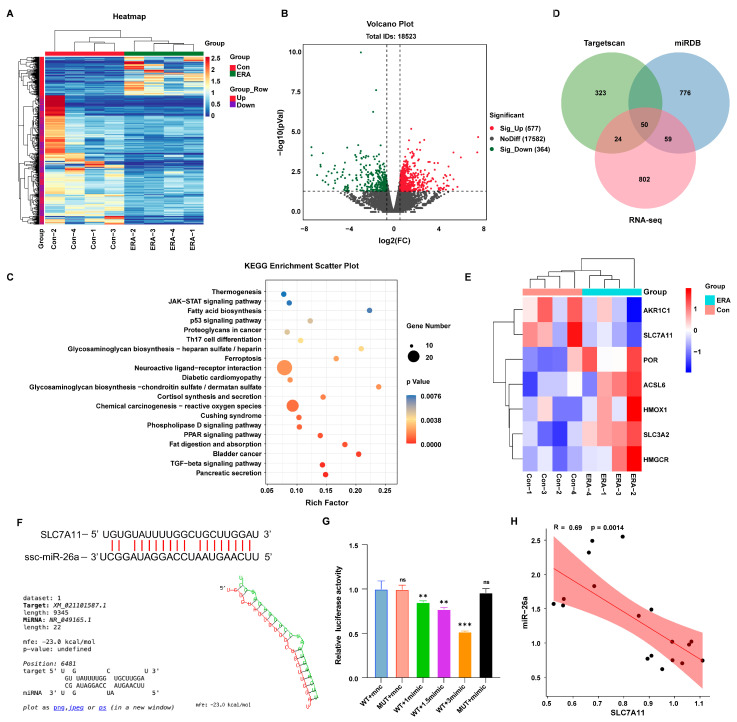
Analysis of differentially expressed genes in erastin-induced follicular ferroptosis. (**A**) Transcriptome sequencing data differential gene clustering heat map; (**B**) Differential gene volcano map of transcriptome sequencing data; (**C**) Venn diagram of target genes and transcriptome differential genes of miR-26a predicted by Targetscan and miRDB; (**D**) Enrichment analysis of sequencing differential miRNAs KEGG pathway; (**E**) Cluster analysis of differential genes associated with ferroptosis in the transcriptome; (**F**) The binding site of miR-26a to SLC7A11 was predicted; (**G**) Dual luciferase reports detected miR-26a binding to SLC7A11, 1mimic means 60 pmol/mL mimics; (**H**) Correlation analysis of miR-26a and SLC7A11. Data are shown as mean ± SD from at least three times biological replicates. ** *p* < 0.01, *** *p* < 0.001, compared to control.

The present findings suggest that miR-26a regulates the onset of ferroptosis in granulosa cells. MicroRNAs can mediate post-transcriptional silencing by binding to complementary mRNA sequences. To further elucidate the mechanism of miR-26a action in granulosa cells, we attempted to find downstream targets of miR-26a. Firstly, we performed transcriptome sequencing on erastin-treated (ERA) cells. Clustering (Figure 4A) and volcano plot (Figure 4B) analyses identified 577 upregulated and 364 downregulated genes compared to controls (Con). Furthermore, KEGG enrichment analysis of these differentially expressed genes indicated involvement in the ferroptosis pathway (Figure 4C). MicroRNAs target mRNAs with complementary sequences to mediate post-transcriptional silencing. Utilizing RNA-seq transcriptome data alongside TargetRank and miRDB analyses, we identified SLC7A11 as a target gene of miR-26a (Figure 4D). Concurrently, clustering heatmap analysis of differentially expressed genes in the erastin-induced ferroptosis pathway revealed significant downregulation of SLC7A11 (Figure 4E). Subsequently, we predicted the binding site of miR-26a to SLC7A11 3′UTR (Figure 4F) and designed mutant sequences against the binding site. These 3′UTR segments were inserted into luciferase reporter plasmids. The luciferase assay revealed a dose-dependent increase in inhibition of luciferase activity for the SLC7A11-WT reporter with rising miR-26a concentrations, while SLC7A11-mutations at the binding site nullified this effect (Figure 4G). Furthermore, miR-26a expression was significantly inversely correlated with SLC7A11 (Figure 4H). These findings confirm that SLC7A11 is a direct target of miR-26a, potentially influencing granulosa cells ferroptosis.

### 3.5. SLC7A11 Negatively Regulates Granule Cell Function After Inducing Ferroptosis

**Figure 5:**
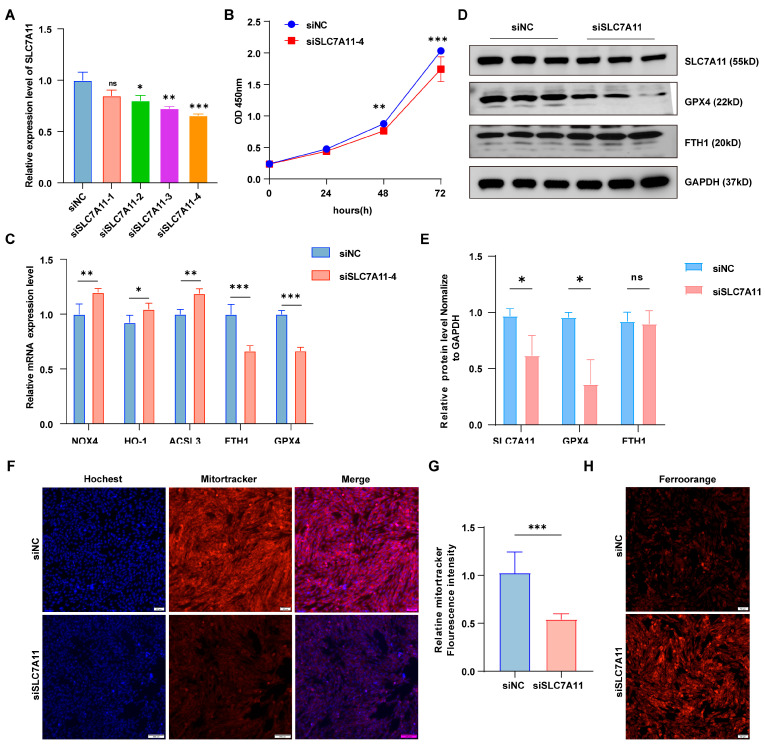
SLC7A11 knockdown inhibited granulosa cell proliferation and promoted ferroptosis. (**A**) Expression of SLC7A11 in four siRNA strands. (**B**) cck-8 detected the viability of cells knockdown SLC7A11. (**C**) qRT-PCR analysis showed the expression levels of iron-toxicity-related genes following SLC7A11 knockdown. (**D**) Western blotting (WB) confirmed protein expression levels, (**E**) presented the WB quantification results. (**F**) Mitochondrial tracking staining assessed mitochondrial activity in siNC and siSLC7A11 groups, with fluorescence quantification results shown in (**G**); Magnification, × 100. (**H**) FerroOrange content of iron ion probe in siNC group and siSLC7A11 group; Magnification, ×100. Data are shown as the mean ± SD of at least three times biological replicates. ns > 0.05, * *p* < 0.05, ** *p* < 0.01, *** *p* < 0.001, compared to control.

SLC7A11, a transmembrane amino acid transporter, plays a crucial role in regulating ferroptosis. To investigate SLC7A11’s role in ferroptosis in granulosa cells, we performed in vitro knockdown of SLC7A11 (Figure 5A). SLC7A11 knockdown significantly reduced cell viability and proliferation (Figure 5B). Furthermore, it markedly increased ferroptosis, evidenced by elevated NOX4, HO-1, and ACSL3 expression, alongside decreased FTH1 and GPX4 levels (Figure 5C). In addition, knockdown of SLC7A11 decreased the protein levels of the ferroptosis marker genes GPX4 and FTH1 (Figure 5D,E), and increased intracellular ferrous ion levels, indicating that SLC7A11 knockdown successfully induced ferroptosis (Figure 5H). Additionally, SLC7A11 knockdown significantly suppressed mitotracker fluorescence intensity and inhibited mitochondrial activity (Figure 5F,G). Collectively, these findings suggest that miR-26a regulates granulocyte ferroptosis through modulation of SLC7A11.

## 4. Discussion

Granulosa cells, a critical cell type surrounding the follicle, can modulate follicular maturation and ovulation [7]. Follicular development relies on granulosa cell proliferation and differentiation, a complex biological process potentially involving miRNAs. Accumulating evidence indicates that miRNA expression levels are closely associated with follicular development and granulosa cell function. Specifically, miR-23a has been identified as pivotal in both normal and pathological processes, including cell cycle regulation, proliferation, and apoptosis [21]. miR-23a and miR-27a both mediated granulosa cell apoptosis by targeting SMAD5, which in turn directly activated the FasL-Fas pathway in vitro [22]. In addition, it has been found that transfection of miR-27a mimics into granulosa cells reduced oocyte maturation rates in mouse follicles [23]. This study investigates the potential regulatory role of a novel form of programmed cell death, ferroptosis, in follicular development. We constructed a ferroptosis model by treating follicles with erastin, a small molecule inducer of ferroptosis [24]. Analysis of miRNA expression in erastin-treated follicles identified miR-26a as significantly upregulated. For the first time, miR-26a is implicated in iron death within follicular granulosa cells, demonstrating its critical role in inhibiting cell proliferation and promoting iron death. Functional assays revealed that miR-26a overexpression significantly suppresses granulosa cell proliferation while concurrently upregulating the expression of iron death markers ACSL4 and PTGS2, along with the apoptosis-iron death crossover molecule Caspase-7. Iron is crucial for activating the iron death pathway. Further analysis showed that miR-26a overexpression increases ferrous ion levels in granulosa cells, suggesting a role in regulating iron ion homeostasis. These findings position miR-26a as a central regulator of iron homeostasis and cell fate in granulosa cells.

Iron-dependent programmed cell death, also known as ferroptosis, is a distinct mode of cell demise characterized by an imbalance in lipid peroxidation and antioxidant defense mechanisms. This dysregulation can impair intracellular cystine transport and glutathione (GSH) synthesis via the cystine/glutamate antiporter SLC7A11 [22,25,26]. The resulting depletion of GSH then induces the expression of key ferroptosis-associated factors, such as PTGS2, ACSL4, and COX2, ultimately leading to cell death. Xia et al. demonstrated that erastin triggers cell death by reducing intracellular glutathione (GSH) levels [27]. Depletion of GSH is a key indicator of ferroptosis and oxidative stress. In this study, erastin treatment reduced GSH levels in granulosa cells. Our mechanistic investigations focused on the cystine/glutamate transporter SLC7A11, crucial for enhancing cellular antioxidant defense. SLC7A11 facilitates cystine uptake, the rate-limiting step in glutathione (GSH) synthesis, which protects against lipid peroxidation and reactive oxygen species (ROS) induced by ferric ion-catalyzed oxidative stress [28]. This indicates that SLC7A11-mediated cystine transport and GSH synthesis are vital for initiating the ferroptosis pathway. We demonstrate that in granulosa cells, miR-26a directly targets the 3′UTR region of SLC7A11, impairing its expression and thereby reducing cellular antioxidant capacity. This was supported by functional experiments, where knockdown of SLC7A11 mimicked the effects of miR-26a overexpression, leading to downregulation of the iron death protective proteins GPX4 and FTH1. Collectively, these findings reveal a granulosa cell iron death pathway triggered by erastin and mediated via the miR-26a/SLC7A11 axis, which provides new insights into the understanding of follicular developmental abnormalities.

Despite these findings, certain limitations of the initial study persist. While we identified the molecular mechanism by which miR-26a targets SLC7A11 in vitro, its role in regulating follicular development and atresia in vivo remains to be verified. Additionally, although we established that erastin upregulates miR-26a, the precise upstream regulatory mechanism remains undefined, warranting further investigation.

In summary, this study elucidates the pivotal role of the miR-26a/SLC7A11 axis in ferroptosis within granulosa cells, offering a foundation for understanding miRNA regulation in ferroptosis during follicular development. Our findings suggest that miRNAs linked to ferroptosis hold promise as diagnostic markers for follicular developmental disorders. While many questions remain, our research underscores the significance of ferroptosis and associated miRNAs in determining follicle fate. Further exploration into the mechanisms governing miRNA biogenesis and function is imperative to elucidate the impact of iron-induced cell death on the broader spectrum of follicular development.

## Data Availability

The original contributions presented in this study are included in the article/Supplementary Material. Further inquiries can be directed to the corresponding author(s).

## Supplementary Materials

The following supporting information can be downloaded at: https://www.mdpi.com/article/10.3390/antiox14111283/s1, Table S1: The primer sequences used for qRT-PCR ( F: forward, R: reverse).
