# Supplementary material for: miR-26a-Targeting SLC7A11 Regulates Erastin-Induced Granulosa Cell Ferroptosis
Source: Antioxidants (Basel). 2025 Oct 26;14(11):1283. doi: 10.3390/antiox14111283 (PMC12649184; doi:10.3390/antiox14111283)
Supplement: Supplementary file 1 [file antioxidants-14-01283-s001.zip › antioxidants-3915130-supplementary.pdf]

Supplemental Materials for

miR-26a targeting SLC7A11 regulates erastin induced granulosa cell ferroptosis

Xue Zhao[1,2,3,4,†], Yuheng Pan[2,3,4,†], Shuang Liang[2,3,4], Yuhang Lei[2,3,4], Yan Wang[2,3,4], Lei Chen[2,3,4], Ye Zhao[2,3,4], Mailin Gan[2,3,4], Linyuan Shen[2,3,4], Xin Yang[1,*], and Li Zhu[2,3,4,*]

† These authors contributed equally to this work.

* Address correspondence to: Xin Yang: yangxin0822@scu.edu.cn and Li Zhu: zhuli@sicau.edu.cn

This word file includes:

Supplementary Table S1

Table S1. The primer sequences used for qRT-PCR (F: forward, R: reverse)

Gene	Primer Sequence (5'-3')	
NOX4	*F*	GAAGGGGTTAAACACCTCTGC
	R	ATGCTCTGCTTAAACACAATCCT
GPX4	*F*	GCCTGGATAAGTACAGGGGTT
	R	CATGCAGATCGACTAGCTGAG
TFRC	*F*	GTTTCTGCCAGCCCCTTATTAT
	R	GCAAGGAAAGGATATGCAGCA
FTH1	*F*	CAAGTGCGCCAGAACTACCA
	R	GCCACATCATCTCGGTCAAAA
FTL	*F*	GAAGATGGGCAACCACCTGA
	R	AGGGCGGGCACGATGTA
ACSL4	*F*	CTCACCATTATATTGCTGCCTGT
	R	TCTCTTTGCCATAGCGTTTTTCT
PTGS2	*F*	TGAGCAACTATTCCAAACCAGC
	R	GCACGTAGTCTTCGATCACTATC
HO-1	*F*	AAGCCGAGAATGCTGAGTTCA
	R	GCCGTGTAGATATGGTACAAGGA
ACSL3	*F*	AACCACGTATCTTCAACACCATC
	R	AGTCCGGTTTGGAACTGACAG
ACTB	*F*	GCTGTATTCCCCTCCATCGT
	R	CTTCTCCATGTCGTCCCAGT
miR-26a	*F*	TTCAAGTAATCCAGGATAGGCT
	R	Uni-miR qPCR Primer, included in kit (miRNA Universal Downstream Primer, TaKaRa)
